# Enhancing the Non-Isothermal Crystallization Kinetics of Polylactic Acid by Incorporating a Novel Nucleating Agent

**DOI:** 10.3390/polym16223204

**Published:** 2024-11-19

**Authors:** Ruijie Jin, Zehong Chen, Yidan Ouyang, Xintu Lin, Xin Dai, Shangxi Zhang, Ruilan Xu, Zhengbao Wang, Yong Peng

**Affiliations:** 1 College of Science, Nanchang Institute of Technology, Nanchang 330099, China; 2 College of Civil and Architecture Engineering, Nanchang Institute of Technology, Nanchang 330099, China; 3 College of Chemical and Biological Engineering, Zhejiang University, Hangzhou 310058, China; 4 Jiangsu Hemoyuan Advanced Materials Technology Co., Ltd., No. 8, Baiyun Road, Nantong 226000, China

**Keywords:** polylactic acid, biochar, multi-walled carbon nanotubes, crystallization rate, non-isothermal crystallization kinetics

## Abstract

Polylactic acid (PLA) is a widely recognized biodegradable polymer. However, the slow crystallization rate of PLA restricts its practical applications. In this study, camphor leaf biochar decorated with multi-walled carbon nanotubes (C@MWCNTs) was prepared using the strong adhesive properties of polydopamine, and PLA/C@MWCNTs composites were fabricated via the casting solution method. The influence of C@MWCNTs as a novel nucleating agent on the melt behavior and non-isothermal crystallization behavior of PLA was investigated using differential scanning calorimetry (DSC). The crystallization kinetic parameters were obtained through the Jeziorny, Ozawa, and Mo methods, and the crystallization activation energy of the PLA/C@MWCNTs composites was calculated by the Kissinger method. The results show that the PLA/C@MWCNTs composites exhibit higher crystallinity and crystallization temperatures than those of PLA. Non-isothermal crystallization kinetic analysis reveals that the Mo method better describes the non-isothermal crystallization kinetics of both PLA and PLA/C@MWCNTs composites. In addition, it was found that C@MWCNTs, despite increasing the crystallization activation energy, can act as an efficient nucleating agent to increase the crystallization rate of PLA. These experimental results provide valuable insights for enhancing the slow crystallization rates associated with PLA.

## 1. Introduction

In recent years, the depletion of petroleum resources and the growing problem of plastic pollution have made biodegradable materials a focus of attention. These materials are increasingly considered as alternatives to conventional petroleum-based polymers in certain fields. Among them, polylactic acid (PLA), recognized for its excellent biodegradability, is considered a promising alternative. Due to its excellent properties, PLA has found extensive applications in packaging, biomedical devices, and fibers [1,2,3]. However, its slow crystallization rate significantly limits its broader adoption and development in certain areas [4,5]. Therefore, improving the crystallization rate of PLA has become essential.

A substantial amount of research has focused on improving the crystallization rate of PLA [6,7,8], with the addition of nucleating agents being recognized as an effective approach [9]. Incorporating nucleating agents can enable PLA to crystallize at higher temperatures during the cooling process [10]. Significant progress has been made in the research of inorganic materials [11,12,13] and many of them have been utilized as nucleating agents to enhance the crystallization process of polylactic acid (PLA), including boron nitride [14,15], aluminum oxide [16], montmorillonite [17,18], graphene [19,20], and multi-walled carbon nanotubes (MWCNTs) [21]. Notably, multi-walled carbon nanotubes (MWCNTs) exhibit a remarkable ability to substantially improve the crystallization rate of PLA. Yousefzade et al. [22] investigated the isothermal and non-isothermal crystallization kinetics of PLA and its nanocomposites with multiwalled carbon nanotubes (pristine and functionalized). They analyzed the effect of nanofiller content on crystallization kinetics and found that the addition of functionalized multiwalled carbon nanotubes at a concentration of 0.2 wt% promoted the crystallization of PLA. Lin et al. [23] synthesized a novel nucleating agent (MWCNTs@CeO_2_) and investigated its effects on the non-isothermal crystallization kinetics of PLA composite films. The results showed that the addition of just 0.7 wt% MWCNTs@CeO_2_ significantly enhanced the crystallization rate of PLA, demonstrating the effectiveness of MWCNTs@CeO_2_ as an effective nucleating agent with high potential. Chen et al. [24] studied the crystallization kinetics of PLA composites reinforced with Kenaf fiber and multi-walled carbon nanotubes. They found that the addition of these fillers significantly enhanced both the crystallization rate and crystallinity of PLA, indicating their effectiveness as nucleating agents.

Camphor trees, regarded as valuable medicinal plants, are plentiful in China. Extracts from camphor leaves have been widely used in pharmaceuticals, food, and cosmetics [25,26]. However, no studies have yet explored the use of biochar derived from camphor leaves as a nucleating agent to modify PLA. Moreover, multifunctional fillers have been shown to significantly enhance the crystallization rate of PLA [27]. Considering the established role of MWCNTs in improving PLA crystallization, this paper investigates the use of MWCNT-modified camphor leaf biochar (C@MWCNTs) as a nucleating agent to prepare PLA-based composites. The objective is to enhance the crystallization properties of PLA and broaden its application fields.

Since PLA processing is primarily carried out under non-isothermal conditions, studying the non-isothermal crystallization kinetics of PLA is of great significance. In this study, MWCNT-modified camphor leaf biochar (C@MWCNTs) was prepared, and C@MWCNTs-modified PLA-based composites were fabricated using the solution casting method. The effects of C@MWCNTs on the non-isothermal crystallization behavior and kinetics of the PLA composites were systematically analyzed.

## 2. Materials and Methods

### 2.1. Materials

The chemical reagents used in this experiment include polylactic acid (PLA, analytical grade, Fengyuan Futailai, Bengbu, China), multi-walled carbon nanotubes (MWCNTs-COOH, analytical grade, Zhongke Times Nano Energy Technology, Chengdu, China), Tris-hydrochloride buffer solution (Tris-HCL, pH = 8.5, analytical grade, Feijing Biotech, Fuzhou, China), and dopamine hydrochloride (analytical grade, Macklin Company, Shanghai, China). HCL, KCl, K_2_CO_3_, and dichloromethane (CH_2_Cl_2_) were purchased from Guangdong Xilong Scientific Co., Ltd., Shantou, China. Camphor leaf biochar (C) was prepared in our laboratory.

### 2.2. Methods

**Scheme 1:**
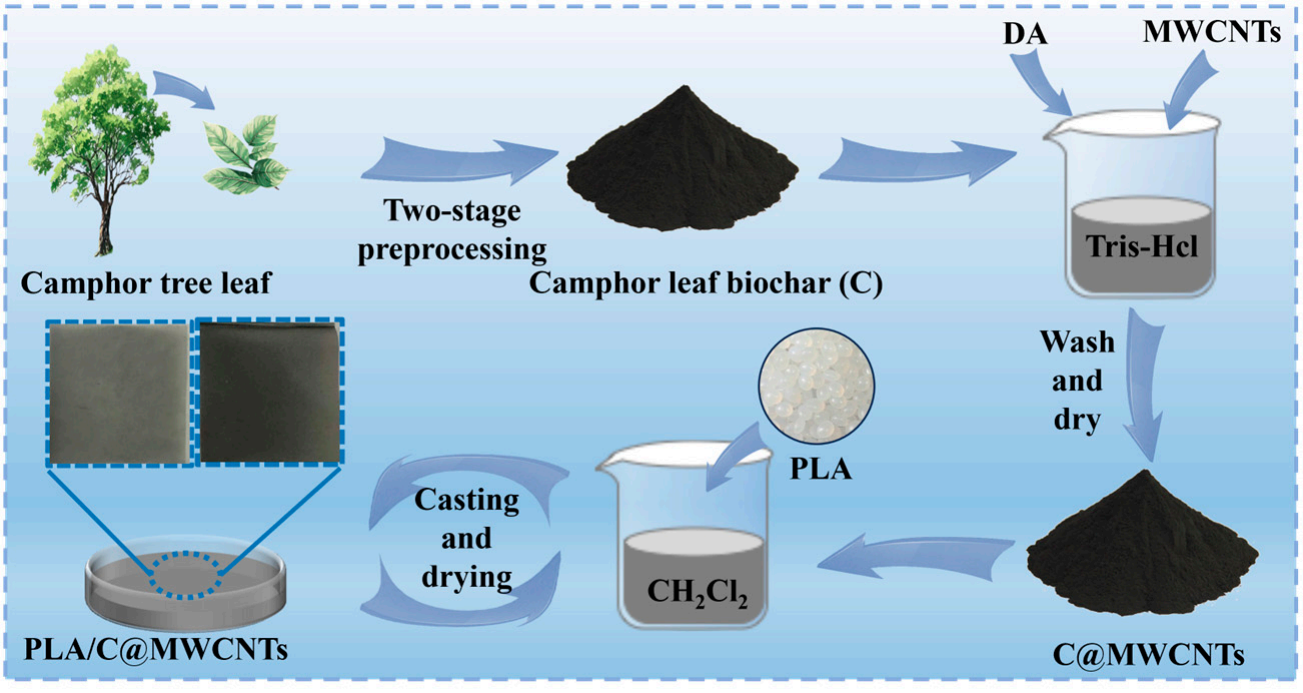
Preparation process of PLA/C@MWCNTs composites.

The preparation process of the PLA/C@MWCNTs composites is shown in Scheme 1.

#### 2.2.1. Preparation of Camphor Leaf Biochar

Camphor leaves were first crushed into a powder and then sieved through a 300-mesh screen to obtain a finer powder. The obtained powders were dried in a drying oven at 60 °C for 12 h. Next, the dried camphor leaf powders were heated from 30 °C to 450 °C at a rate of 5 °C/min in a muffle furnace, and then kept at this temperature for 30 min. Subsequently, the obtained powders were cooled to room temperature. The pre-carbonized powders were then soaked in a saturated solution of potassium chloride and potassium carbonate (KCl/K_2_CO_3_) for 12 h, with continuous stirring, followed by drying in an oven at 80 °C. Subsequently, the dried powders were placed in a furnace and calcined in a nitrogen atmosphere. The temperature was raised to 900 °C at a rate of 5 °C/min and kept at this level for 90 min, after which it was cooled to room temperature. The obtained powders were then immersed in a 1 M hydrochloric acid (HCL) solution for 1 h, followed by thorough washing with deionized water and anhydrous ethanol. Finally, the the camphor leaf biochar (C) was obtained after drying in an oven at 60 °C for 12 h.

#### 2.2.2. Preparation of C@MWCNTs

Next, 0.2 g of camphor leaf biochar and 0.2 g of dopamine hydrochloride were dissolved in 80 mL of Tris-hydrochloride buffer solution, and the mixture was stirred magnetically for 2 h to obtain a homogeneous solution. Subsequently, 0.2 g of carboxylated multi-walled carbon nanotubes (MWCNTs-COOH) was added to the solution, which was then stirred magnetically for an additional 2 h and subsequently filtered. The resulting product was washed several times with deionized water and anhydrous ethanol, and then dried at 60 °C for 12 h to obtain the camphor leaf biochar@multi-walled carbon nanotubes (C@MWCNTs).

#### 2.2.3. Preparation of PLA and PLA/C@MWCNTs Composites

A specific amount of C@MWCNTs was dispersed in 20 mL of dichloromethane (CH_2_Cl_2_) using ultrasonication for 30 min to obtain a uniform dispersion. An appropriate amount of PLA was then added to the dispersion, followed by ultrasonication for 1.5 h and magnetic stirring for 3 h. After ultrasonication for 5 min to remove air bubbles, the mixture was poured into a polytetrafluoroethylene (PTFE) mold and allowed to dry naturally for 12 h. Finally, the PLA/C@MWCNTs composites containing 0 wt%, 0.1 wt%, and 0.3 wt% were prepared and designated as PLA, PLA/0.1%C@MWCNTs, and PLA/0.3%C@MWCNTs, respectively.

#### 2.2.4. Characterization of Non-Isothermal Crystallization

The non-isothermal crystallization behavior and kinetics of the samples were investigated using a differential scanning calorimeter (DSC 25, TA, New Castle, DE, USA). Under a nitrogen atmosphere, samples weighing 5–8 mg were heated from 30 °C to 200 °C at a rate of 20 °C/min and held at 200 °C for 3 min to eliminate any thermal history, then cooled to 30 °C at varying rates of 5, 10, 15, and 20 °C/min, and subsequently reheated to 200 °C at the same heating rate. The crystallinity of PLA during the crystallization process was calculated using Equation (1):

(1) $$X_{c}=\frac{∆H_{m}-∆H_{cc}}{w_{f}{∆H}_{m}^{0}}\times 100\%$$

where ∆H_m_ denotes the melting enthalpy, ∆H_cc_ is the cold crystallization enthalpy, and w_f_ refers to the mass percentage of PLA in the composites. ${∆H}_{m}^{0}$ represents the melting enthalpy of 100% crystalline PLA, which is defined as 140 J/g [28].

## 3. Results and Discussion

### 3.1. Effects of C@MWCNTs on Melting and Crystallization Behavior of PLA

**Figure 1:**
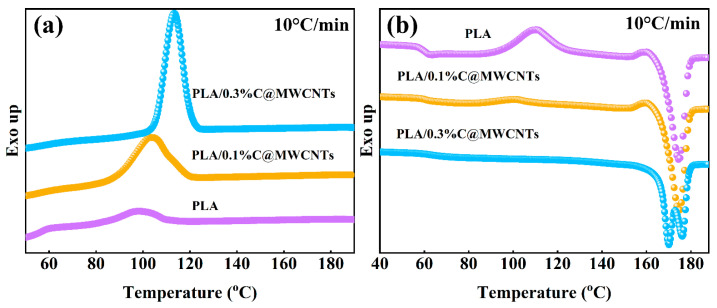
Cooling curve (**a**) and second heating curve (**b**) of PLA with different C@MWCNTs contents.

Figure 1 illustrates the melting and crystallization behaviors of the PLA, PLA/0.1%C@MWCNTs, and PLA/0.3%C@MWCNTs composites. From Figure 1a, it can be observed that the crystallization peak temperature of pure PLA is 97.5 °C and features a broad peak. With the increase in C@MWCNTs content, the crystallization peak not only shifts to higher temperatures, but also becomes sharper, demonstrating that C@MWCNTs function as heterogeneous nucleating agents. This enhancement facilitates the nucleation and crystallization of PLA, enabling it to crystallize at elevated temperatures during the cooling process. As shown in Figure 1b, cold crystallization peaks were only observed in the DSC heating curves of pure PLA and PLA with 0.1%C@MWCNTs. As we all know, cold crystallization takes place during heating from the glassy state [29]. When the C@MWCNTs content reaches 0.3 wt%, the cold crystallization peak of PLA disappears, indicating that the addition of C@MWCNTs enhances the crystallization rate and ability of PLA, resulting in more complete crystallization. In addition, when the C@MWCNTs content increases from 0 wt% to 0.3 wt%, the crystallinity of PLA increases from 16.35% to 28.81%, representing an improvement of 76.21%. Liang et al. [30] found that the addition of 0.1% fluorinated CNTs (CNTs-F) increased the crystallinity of PLA from 5% to 8%, representing a 60% improvement. Compared to CNTs-F, the nucleating agent (C@MWCNTs) used in this study seems to be more beneficial for enhancing the crystallization behavior of PLA. As seen in Figure 1b, the melting curves of pure PLA and PLA containing 0.1% C@MWCNTs each display a single melting peak, while the melting process of PLA containing 0.3% C@MWCNTs exhibits bimodal melting behavior. Moreover, the higher temperature melting peak of PLA with 0.3% C@MWCNTs shifts to a higher temperature when compared to the melting peak of pure PLA. These results suggest that the incorporation of C@MWCNTs improves the crystallization of PLA.

### 3.2. Non-Isothermal Crystallization Kinetics of PLA/C@MWCNTs Composites

To investigate the effect of C@MWCNTs on the overall crystallization rate of PLA, the non-isothermal crystallization kinetics of PLA and PLA/C@MWCNTs composites were studied. In non-isothermal crystallization, the relationship between the relative crystallinity (X_t_) and temperature (T) can be expressed using Formula (2) [23,31]:

(2) $$X_{t}=\int_{T_{0}}^{T}\left(\frac{{dH}_{c}}{dT}\right)dT/\int_{T_{0}}^{T_{\infty }}\left(\frac{{dH}_{c}}{dT}\right)dT$$

where T_0_ refers to the onset temperature of the crystallization process, $T_{\infty }$ represents the end crystallization temperature, and dH_c_ is the enthalpy change associated with crystallization that occurs over an infinitesimal temperature interval, dT. The crystallization time (t) can be determined based on the crystallization temperature, as expressed in Equation (3):

(3) $$t=\left(T_{0}-T\right)/\beta$$

where T represents the temperature at crystallization time t, and β is the cooling rate.

**Figure 2:**
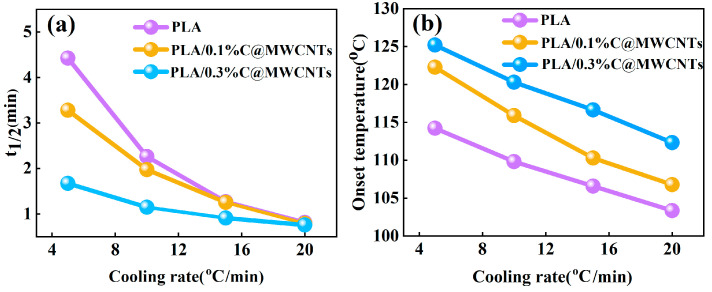
Plots of cooling rate versus (**a**) t_1/2_ value and (**b**) onset temperature for PLA/C@MWCNTs composites.

The semi-crystallization time (t_1/2_) refers to the duration required to reach 50% of the final crystallinity of the sample, which can be directly obtained from the plot of relative crystallinity versus crystallization time. Figure 2 shows the variations in the t_1/2_ values and onset temperatures of the PLA and PLA/C@MWCNTs composites at various cooling rates. As depicted in Figure 2a, with increasing cooling rates, the t_1/2_ values of the PLA/C@MWCNTs composites decrease, indicating that higher cooling rates promote nucleation and crystallization growth in the composites. At a given cooling rate, pure PLA exhibits the highest t_1/2_ value. In addition, as the content of C@MWCNTs increases, the t_1/2_ values gradually decrease, with PLA/0.3%C@MWCNTs showing the lowest t_1/2_ value. The decrease in t_1/2_ indicates that the incorporation of C@MWCNTs into PLA enhances and speeds up the crystallization process. Based on the observations in Figure 2b, it is evident that as the crystallization rate increases, the onset crystallization temperature decreases. Furthermore, at the same crystallization rate, the onset crystallization temperature of PLA/0.3%C@MWCNTs is the highest, while PLA exhibits the lowest onset crystallization temperature. This further confirms that the addition of C@MWCNTs promotes the crystallization of PLA.

The Avrami equation [32,33,34] is commonly used to describe the isothermal crystallization process of polymers by using the following Formulas (4) and (5):

(4) $${1-X}_{t}=exp\left(-Zt^{n}\right)$$

(5) $$\log\left[-\ln\left({1-X}_{t}\right)\right]=\log Z+n\log t$$

where n is the Avrami exponent, Z is the crystallization rate constant, and X_t_ is the relative crystallinity at time t.

For the non-isothermal crystallization, the crystallization rate constant Z was modified by the Jeziorny method [35] to accommodate the non-isothermal crystallization process using Formula (6):

(6) $$\log Z_{c}=\left(\log Z\right)/\beta$$

where Z_c_ is the corrected crystallization rate constant, and β refers to the cooling rate.

**Figure 3:**
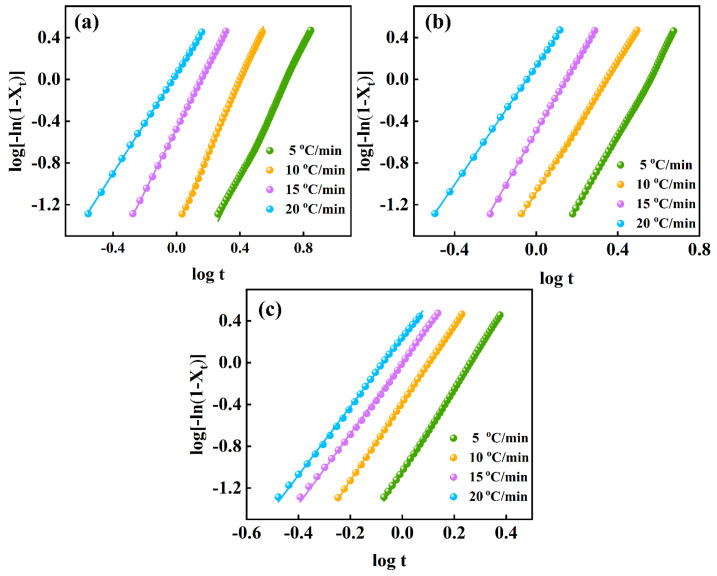
Plots of $\log\left[-\ln\left({1-X}_{t}\right)\right]$ as function of log t for (**a**) PLA, (**b**) PLA/0.1%C@MWCNTs, and (**c**) PLA/0.3%C@MWCNTs composites.

Figure 3 shows the relationship between log[−ln(1 − X_t_)] and log t (with relative crystallinity ranging from 5% to 95%) for the PLA and PLA/C@MWCNTs composites at different cooling rates. The results obtained from the slopes and intercepts of these curves are presented in Table 1. As seen in Table 1, a strong linear relationship (r^2^ > 0.997) exists between log[−ln(1 − X_t_)] and log t for all samples. The n values for pure PLA range from 2.39 to 3.51; for PLA/0.1%C@MWCNTs, from 2.84 to 3.50; and for PLA/0.3%C@MWCNTs, from 3.28 to 3.97, indicating that the crystallization mechanism of PLA and its composites undergoes three-dimensional spherical growth during non-isothermal crystallization [34]. In addition, the Z_c_ values for all samples increase with increasing cooling rates, demonstrating that the non-isothermal crystallization rate increases as the cooling rate increases. At a given cooling rate, the Z_c_ values adhere to the following order: PLA/0.3%C@MWCNTs > PLA/0.1%C@MWCNTs > PLA, indicating a higher crystallization rate for PLA composites containing C@MWCNTs, consistent with the t_1/2_ results. Although Jeziorny applied Avrami’s equation to describe non-isothermal crystallization by introducing a correction factor, this approach has certain limitations, as highlighted in a recent study by Vyazovkin [36].

**Figure 4:**
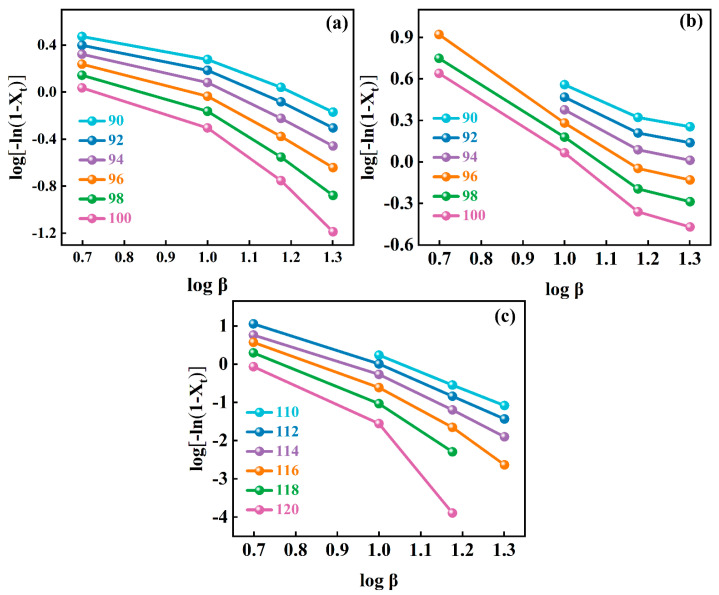
Plots of $\log\left[-\ln\left({1-X}_{t}\right)\right]$ as function of log⁡β for (**a**) PLA, (**b**) PLA/0.1%C@MWCNTs, and (**c**) PLA/0.3%C@MWCNTs composites.

The Ozawa method extended the Avrami equation to non-isothermal crystallization by considering the effect of cooling rate on the polymer crystallization process. Assuming that the non-isothermal crystallization process consists of infinitesimal isothermal crystallization steps leads to the following Equations (7) and (8) [37,38]:

(7) $$X_{t}=1-exp\left(-\frac{K\left(T\right)}{\beta^{m}}\right)$$

or

(8) $$\log\left[-\ln\left({1-X}_{t}\right)\right]=\log K\left(T\right)-m\log\beta$$

where K(T) denotes the cooling function associated with the crystallization rate, and m represents the Ozawa exponent. To facilitate analysis, plots depicting log[−ln(1 − Xt)] against log⁡β were generated for the PLA and PLA/0.1%C@MWCNTs composite systems over a temperature range of 90–100 °C. Additionally, the PLA/0.3%C@MWCNTs system was examined within the temperature range of 110–120 °C, as illustrated in Figure 4. Based on Figure 4, it is evident that no straight lines are present, suggesting that the Ozawa model does not effectively describe the non-isothermal crystallization behavior of pure PLA and its composite materials. These results may be attributed to the adaptation of Avrami’s equation in the Ozawa model, which consistently neglects the influences of the secondary crystallization process [31].

Mo et al. [39] combined the Avrami and Ozawa equations to propose a novel approach, resulting in Equations (9) and (10):

(9) $$\log Z+\mathrm{nlog}t=\log K\left(T\right)-m\log\beta$$

(10) $$\log\beta =\log F\left(T\right)-\alpha \log t$$

where F(T) = [K(T)/Z]^1/m^ denotes the cooling rate required for a system to reach a specific degree of crystallinity within a unit of time, and α represents the ratio of the Avrami exponent (n) to the Ozawa exponent (m).

**Figure 5:**
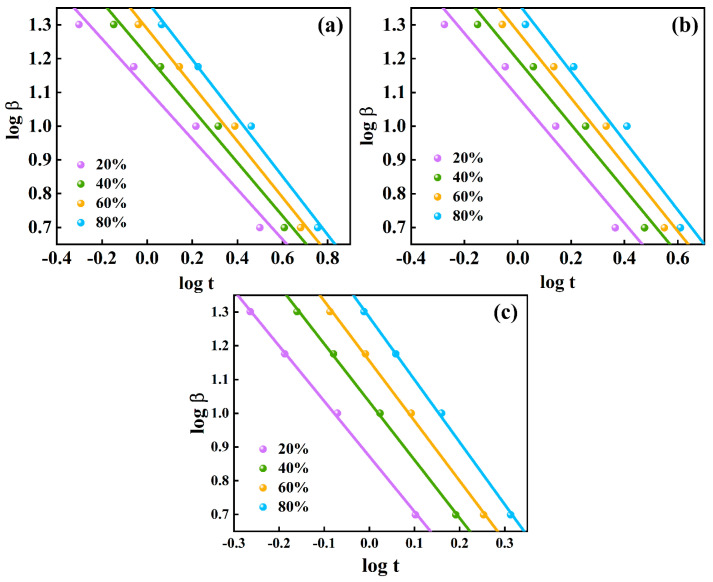
Plots of log⁡β versus log⁡t for non-isothermal crystallization of (**a**) PLA, (**b**) PLA/0.1%C@MWCNTs, and (**c**) PLA/0.3%C@MWCNTs composites.

Figure 5 presents the plots of log⁡β versus log t (with relative crystallinity ranging from 20% to 80%) for the PLA and PLA/C@MWCNTs composites. Notably, the linearity of these curves suggests that the Mo method accurately describes the non-isothermal crystallization behavior of PLA and its composites. By analyzing the slopes and intercepts of the fitted curves, the values of F(T) and α were derived, as detailed in Table 2. It can be seen that the α values for PLA range from 0.743 to 0.865, while those for PLA-0.1%C@MWCNTs range from 0.938 to 1.024, and for PLA/0.3%C@MWCNTs, the values range from 1.637 to 1.849. This result indicates that the addition of MWCNTs@Ag may affect the nucleation and crystal growth mechanisms of PLA. Meanwhile, the values of F(T) increase with rising relative crystallinity, indicating that PLA’s crystallization becomes increasingly difficult as its crystallinity increases, which may lead to the termination of crystallization or the ordering of flexible, linear macromolecules before completion. In addition, at the same level of relative crystallinity, PLA exhibits the highest F(T) values, while PLA/0.3%C@MWCNTs presents the lowest. These results further support the idea that incorporating MWCNTs@Ag into PLA enhances the crystallization process. This finding is consistent with the results from the Jeziorny equation analysis and t_1/2_.

### 3.3. Crystallization Activation Energy of PLA/C@MWCNTs Composites

The Kissinger formula is commonly used to evaluate the effective activation energy of the non-isothermal crystallization of polymers, and it can be expressed using Equation (11) [10], as follows:

(11) $$\frac{d\left[\ln\left(\beta /T_{p}^{2}\right)\right]}{d\left(1/T_{p}\right)}=-\frac{∆E}{R}$$

where β denotes the cooling rate, ∆E represents the activation energy of crystallization, T_p_ is the peak crystallization temperature, and R is the gas constant.

**Figure 6:**
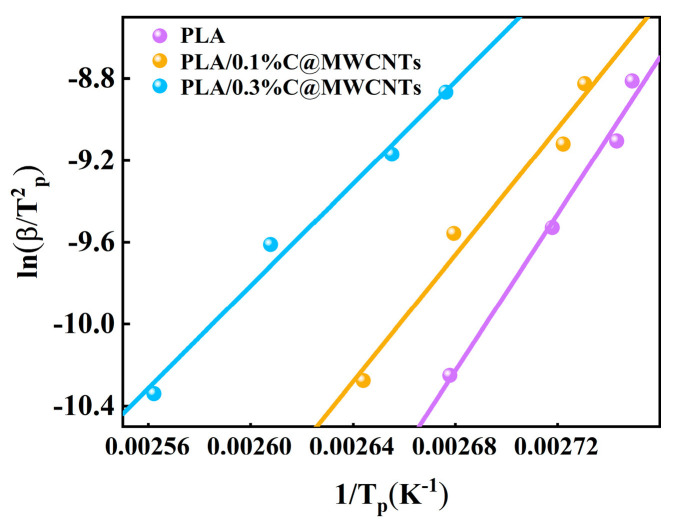
Plots of $ln(\beta /T_{p}^{2})$ versus $1/T_{p}$ for PLA /C@MWCNTs composites.

By plotting $ln(\beta /T_{p}^{2})$ versus 1/T_p_ and performing linear fitting, the activation energy of each sample can be obtained from the slope of the fitted curve. Figure 6 shows the fitted curves of $ln(\beta /T_{p}^{2})$ versus 1/T_p_ for the PLA composites. The activation energy values of the PLA, PLA/0.1%C@MWCNTs, and PLA/0.3%C@MWCNTs composites were calculated to be −159.00 kJ/mol, −128.50 kJ/mol, and −103.86 kJ/mol, respectively. Clearly, the PLA/C@MWCNTs composites exhibit higher crystallization activation energy than pure PLA, suggesting that the addition of C@MWCNTs increases the energy barrier for PLA crystallization. This is likely due to the reduced mobility of the PLA molecular chains caused by the incorporation of C@MWCNTs, which hinders their rearrangement. A similar phenomenon was reported by Zhao et al. [40] in their study on the non-isothermal crystallization kinetics of PLA.

## 4. Conclusions

In this study, camphor leaf biochar decorated with multi-walled carbon nanotubes (C@MWCNTs) was prepared and utilized as a nucleating agent for PLA. PLA-based composites containing different contents of C@MWCNTs were prepared using the solution casting method. The non-isothermal crystallization behavior of PLA nucleated by C@MWCNTs was investigated by differential scanning calorimetry (DSC), and the corresponding non-isothermal crystallization kinetics parameters were analyzed using the Jeziorny method, Ozawa, and Mo models. The results indicate that the addition of C@MWCNTs increases the crystallization temperature (T_c_) and the crystallinity of PLA. Non-isothermal crystallization experiments demonstrate that C@MWCNTs serve as a potent nucleating agent, enhancing the crystallization rate of PLA during non-isothermal processes. This effectiveness is further confirmed by several non-isothermal crystallization parameters including semi-crystallization time (t_1/2_), modified crystallization rate constant, and F(T). In addition, the Kissinger method was employed to determine the activation energy of PLA, and it is observed that the addition of C@MWCNTs results in an increase in activation energy.

## Figures and Tables

**Table 1:** Avrami and Jeziorny parameters of PLA/C@MWCNTs composites.

Samples	β (°C/min)	n	Z_c_	r^2^
PLA	5	3.13	0.367	0.99787
	10	3.51	0.722	0.99904
	15	3.00	0.930	0.99995
	20	2.39	1.007	0.99982
PLA/0.1% C@MWCNTs	5	3.50	0.411	0.99811
	10	3.13	0.780	0.99961
	15	3.40	0.926	0.99967
	20	2.84	1.015	0.99992
PLA/0.3% C@MWCNTs	5	3.97	0.618	0.99959
	10	3.71	0.915	0.99993
	15	3.38	1.000	0.99888
	20	3.28	1.028	0.99931

Notes: β is the cooling rate; n is the Avrami exponent; Z_c_ is the modified crystallization rate constant; r^2^ is the linear correlation coefficient.

**Table 2:** Non-isothermal crystallization kinetic parameters for PLA/C@MWCNTs composites at different relative crystallinities based on Mo equation.

Samples		Relative Crystallinities (%)			
		20	40	60	80
PLA	α	0.743	0.793	0.832	0.865
	F(T)	12.886	16.202	19.421	23.455
PLA/0.1%C@MWCNTs	α	0.938	0.958	0.986	1.024
	F(T)	12.242	15.735	19.077	23.227
PLA/0.3%C@MWCNTs	α	1.637	1.720	1.777	1.849
	F(T)	7.449	10.804	14.294	19.243

Notes: α is the ratio of the Avrami exponent to the Ozawa exponent; F(T) represents the required cooling rate for the polymer system to achieve a specific level of crystallinity within a defined crystallization time.

## Data Availability

The data presented in this study are available on request from the corresponding author due to privacy.
